# Optimization meets systems biology

**DOI:** 10.1186/1752-0509-4-S2-S1

**Published:** 2010-09-13

**Authors:** Yong Wang, Xiang-Sun Zhang, Luonan Chen

**Affiliations:** 1 Academy of Mathematics and Systems Science, Chinese Academy of Sciences, Beijing 100190, China; 2 Key Laboratory of Systems Biology, SIBS-Novo Nordisk Translational Research Centre for PreDiabetes, Shanghai Institutes for Biological Sciences, Chinese Academy of Sciences, Shanghai 200031, China

## Abstract

A report of the 3nd International Symposium on Optimization and Systems Biology, 20-22 September 2009, Zhangjiajie, China.

## Background

Optimization, in particular mathematical programming, refers to minimizing or maximizing a real function by systematically choosing the values of real or integer variables from a feasible set mathematically. More generally, it means finding "best available" values of some objective function given a defined domain, including a variety of different types of objective functions and domains. Optimization methods are among the most commonly used mathematical tools in scientific research. This is because they are well-defined and easy to be understood once an objective function and its constraint are chosen.  It’s well known that optimization methods have been widely applied in biological research [1-3]. For example, the protein folding problem is a fundamental important problem and is a typical example for the interplay between biology, chemistry, and optimization techniques [4]. Regarding to the fact that current biological structures and functions are optimized by evolution over a long time, optimization is a natural and powerful tool for modeling complex biological problems. 

Compared to optimization, systems biology is a newly proposed term to describe the study of the interactions between the components of biological systems, and how these interactions give rise to the function and behavior of that system. With increasingly accumulated data from high-throughput technologies, molecular networks and their dynamics have been studied extensively from various aspects of living organisms. Many mathematical methods have been adopted in computational systems biology; in particular, optimization plays a key role in analysing and understanding biological mechanisms from system-wide viewpoints.  For example, linear programming and convex programming have been widely developed to reconstruct gene regulatory network, transcriptional regulatory network, protein interaction network, conditional specific subnetworks, and active pathways [5].  Quadratic programming has been applied to align two different types of biomolecular networks [5], and optimization has been the main engine behind metabolic flux balance analysis in metabolic networks [5]. In addition, examples are given in a recent review where optimization methods are used for topics ranging from model building and optimal experimental design to metabolic engineering and synthetic biology [6].

However, the development of novel optimization methodologies for systems biology is still in its infancy and much research remains to be done in this area. There is a lot of room for further improvement of the existing methods by employing advanced optimization theory and algorithm design techniques. More importantly, many practical problems in systems biology will further challenge the current optimization methods and accelerate the development on new theory and algorithm.  OSB series symposium is interdisciplinary by its nature, and focuses on bridging opportunities between Optimization and Systems Biology studies.

## Meeting report

A three-day international symposium on Optimization and Systems Biology was held in 20-22 September, 2009 (OSB2009)  in Zhangjiajie, which is a must-see UNESCO World Heritage area known as world geological park in central-south China. More than 100 researchers including engineers, mathematicians and biologists from China mainland, United States, Hong Kong, Taiwan, Japan, Korea, Singapore, India, Portugal, Australia, and Poland enjoyed both academic exchanges and natural scenes.

Following the successful OSB 2007(http://www.aporc.org/ISB/2007/index.php )  and OSB 2008 (http://www.aporc.org/ISB/2008/index.php), the purposes of OSB 2009 is to extend the international forum for scientists, researchers, educators, and practitioners to exchange their ideas by presenting research findings and state-of-the-art solutions in this interdisciplinary field, including optimization methods and its applications in biosciences and researches on various aspects of Systems Biology.  

The Proceedings of the Third International Symposium on Optimization and Systems Biology (OSB 2009) have been published by World Publishing Corporation (ISBN 978-7-5100-0549-7/O764) as Lecture Notes in Operations Research 11 and the proceedings are freely available online (http://www.aporc.org/LNOR/11/). Fifty nine papers in this volume cover wide range of optimization and systems biology and all the papers are indexed by ISTP (Index to Scientific \& Technical Proceedings). Moreover, the reviewers from the Program Committee of OSB2009 selected 13 papers for a special issue in BMC Systems Biology after significant extension of their versions on the Proceedings.  Each submission has been peer reviewed and evaluated by three independent reviewers on the quality, originality, soundness, and significance of its contributions and the significant improvement regarding to the OSB proceeding paper. Here we focus on some of the highlights of the meeting by categorizing and briefly introducing these selected papers.

## Reconstruction and analysis of biomolecular networks

The key research objects of systems biology are biomolecular networks. The successful bio-technologies enable us to simultaneously measuring the concentrations of thousands of biomolecules. Such high throughput data offer great chances to reconstruct biomolecular networks by searching the network structure and parameters to optimize some criteria. For example, one can choose a model to maximize its consistence with the experimental data or to optimize some design principles of biological networks, such as making the network sparse to better fit the limited biological data.

Binhua Tang et al. proposed a supervised combinatorial-optimization pattern based on information and signal processing theories to infer and analyze the genetic regulatory networks. Firstly, an associativity measure was proposed to define the regulatory strength/connectivity, and then a phase shift metric was adopted to determine regulatory directions among components of the reconstructed networks. Furthermore they gave a condition to restrict the classified group size of pair candidates within a multiobjective combinatorial optimization (MOCO) pattern.

For the transcriptional regulatory network reconstruction, Gyan Prakash Srivastava et al. used a novel approach that combines kinetic modelling of gene expression with a statistical meta-analysis to predict targets of 757 TFs using expression data of 14,905 genes in Arabidopsis exposed to different durations and types of abiotic stresses. Using a kinetic model for the time delay between the expression of a TF gene and its potential targets, they shifted a TF’s expression profile to make an interacting pair coherent. It was found that partitioning the expression data by tissue and developmental stage improved correlation between TFs and their targets. 

To optimally integrate heterogeneous data sources, Fei Luo et al. proposed a framework of discovering the conditional co-regulated protein complexes. The method was tested on the Yeast data sets under the Cell Cycle, DNA Damage and Dauxic Shift conditions, and identified a total of 32 conditional co-regulated complexes, among which the coding genes in 16 complexes show a strong association with their TFs activity. Based on the close relationship among co-regulation, co-expression and protein-protein interactions in the conditional co-regulated protein complexes, 36 novel TRs were predicted and explained.

Recent development of high-resolution single-nucleotide polymorphism (SNP) arrays allows the reconstruction of SNP networks.  Yang Liu et al. used shrunken dissimilarity measure to analyze and select relevant SNPs. They used Parkinson disease data as an example, and performed a whole genome analysis. For the 367440 SNPs with less than 1% missing percentage from all 22 chromosomes, in total 357 SNPs were selected from this data set. For the unique genes that those SNPs were located in, a gene-gene similarity value was computed using GOSemSim and gene pairs that has a similarity value being greater than a threshold were selected to construct several groups of genes. For the SNPs that were involved in these groups of genes, a statistical software PLINK was employed to compute the pair-wise SNP-SNP interactions, and SNPs with significance of P < 0.01 were chosen to identify SNPs networks based on their P values. Here SNPs networks were constructed based on Gene Ontology knowledge, and therefore each SNP network plays a role in the biological process. An analysis shows that such networks have relationships directly or indirectly to Parkinson disease.

## Predicting drug targets and drug combination

Predicting drug-protein interactions from heterogeneous biological data is a key step for in-silico drug discovery. To meet this challenge, Zheng Xia et al. proposed a manifold regularization semi-supervised learning method to integrate known drug-protein interaction network information as well as chemical structure and genomic sequence data. Using the proposed method, they gave encouraging results on drug-protein interaction network reconstruction and predicted certain drug-protein interactions on the enzyme, ion channel, GPCRs, and nuclear receptor data sets, which may shed light on the molecular interaction inference and new uses of marketed drugs.

Instead of  drug targets, Zikai Wu et al. emphasized combination regimens or combination drugs, which provide an alternative way to combat complex diseases, and are becoming the standard of treatment for complex diseases. Actually, most of existing combination drugs were developed based on clinical experience or test-and-trial strategy, which are not only time consuming but also expensive. Then they presented a novel network-based systems biology approach to identify effective drug combinations by exploiting high throughput data. Specifically, they first constructed a molecular interaction network by integrating protein interactions, protein-DNA interactions, and signaling pathways. A new model was then developed to detect subnetworks affected by drugs. Furthermore, a new score was designed to evaluate the overall effect of one drug by taking into account both efficacy and side-effects. The proposed method was applied to identify effective combinations of drugs used to treat Type 2 Diabetes, and detected the combination of Metformin and Rosiglitazone, which is actually Avandamet, a drug that has been successfully used to treat Type 2 Diabetes.

Drug combination is well-known for its importance in traditional Chinese medicine. Hao-Teng Chang et al. experimentally tested eighty-three Chinese herbs and prescriptions, and five effective herbs and six prescription candidates were selected. On the basis of effective single-herbal drugs and prescriptions, a combinative network was generated. They found that a single herb, Gan-cao, served as a node connecting five prescriptions. In addition, Sheng-di-huang, Dang-guei and Mu-tong also appeared in five, four and three kinds of prescriptions, respectively. The extracts of these three herbs indeed effectively inhibited the interactions between ECP and Beas-2B cells. According to the Chinese herbal combinative network, eight of the effective herbal extracts showed inhibitory effects for ECP internalizing into Beas-2B cells. The major components of Gang-cao and Sheng-di-huang, glycyrrhizic acid and verbascose, respectively, reduced the binding affinity between ECP and cells effectively.

## Subnetwork study in biomolecular networks

The investigation on network dynamics through the subnetworks is a major issue in systems and synthetic biology. Both the identification of subnetwork structure and parameter estimation require the design or implementation of  optimization models.

Masahiko Nakatsui et al. proposed a new approach for parameter optimization by using differential elimination, to estimate kinetic parameter values with a high degree of accuracy. First, they utilized differential elimination, which is an algebraic approach for rewriting a system of differential equations into another equivalent system, to derive the constraints between kinetic parameters from differential equations. Second, they estimated the kinetic parameters converting these constraints into an objective function, in addition to the error function of the square difference between the measured and estimated data, in the standard parameter optimization method. To evaluate the ability of the method, they performed a simulation study by using the objective function with and without the newly developed constraints: the parameters in two models of linear and non-linear equations, under the assumption that only one molecule in each model can be measured, were estimated by using a genetic algorithm (GA) and particle swarm optimization (PSO). 

Cellular functions and biochemical events are coordinately carried out by subnetworks of proteins interacting each other in biological modules. Shihua Zhang et al contributed a paper to identify such modules in protein interaction networks, which is very important for understanding the structure and function of these fundamental cellular networks. Therefore, they introduced a new quantitative measure modularity density and developed new algorithms for detecting functional modules in protein-protein interaction (PPI) networks. Specifically, they adopted the simulated annealing (SA) to maximize the modularity density and evaluate its efficiency on simulated networks. 

Zhi-Ping Liu et al. proposed a network-based systems biology approach to detect the crosstalks among Alzheimer’s disease (AD) related pathways, as well as their dysfunctions in the six brain regions of AD patients. Through constructing a network of pathways, the relationships among AD pathway and its neighbor pathways were systematically investigated and visually presented by their intersections. They found that the significance degree of pathways related to the fatal disorders and the pathway overlapping strength can indicate the impacts of these neighbored pathways to AD development. Furthermore, it was shown that the neighbor pathways of the AD pathway closely cooperate and play important tasks in the AD progression.

## Optimization methods for systems biology

As mentioned earlier, optimization is a powerful tool to solve the systems biology problems. For example, Chenglei Sun et al. used an optimization method, i.e., support vector machine, to predict F. graminearum protein subcellular localizations from the primary structures. First, a non-redundant fungi data set with subcellular localization annotation was collected from UniProtKB database and used as a training set, where the subcellular locations were classified into 10 groups. Subsequently, Support Vector Machine (SVM) was trained on the training set and used to predict F. graminearum protein subcellular localizations for those proteins that do not have significant sequence similarity to those in training set. 

Morihiro Hayashida  et al. proposed novel efficient methods, i.e., CompressEdge and CompressVertices, for comparing large biological networks. In the proposed method, an original network structure was compressed by iteratively contracting identical edges and sets of connected edges. Then, the similarity of two networks was measured by a compression ratio of the concatenated networks. The proposed method was applied to comparison of metabolic networks of several organisms, H. sapiens, M. musculus, A. thaliana, D. melanogaster, C. elegans, E. coli, S. cerevisiae, and B. subtilis, and was also compared with an existing method. 

Probabilistic Boolean Networks (PBNs) provide a convenient tool for studying gene regulatory networks.  Cong Yang et al. studied the intervention problem by manipulating multiple external controls in a finite time interval in a PBN. The maximum numbers of times that each control method can be applied were given. They treated the problem as an optimization problem with multi-constraints and introduced an algorithm, the “Reserving Place Algorithm”, for finding all optimal intervention strategies. Given a fixed number of times that a certain control method is applied, the algorithm can provide all the sub-optimal control policies. Theoretical analysis for the upper bound of the computational cost was also given. They also developed a heuristic algorithm based on Genetic Algorithm, to find the optimal intervention strategy for networks with large sizes.

## Competing interests

The authors declare that they have no competing interests.

## Authors' contributions

YW drafted the manuscript. XSZ and LC read and approved the manuscript.
